# Ethical AI in Healthcare: Integrating Zero-Knowledge Proofs and Smart Contracts for Transparent Data Governance

**DOI:** 10.3390/bioengineering12111236

**Published:** 2025-11-12

**Authors:** Mohamed Ezz, Alaa S. Alaerjan, Ayman Mohamed Mostafa

**Affiliations:** 1 Department of Computer Science, College of Computer and Information Sciences, Jouf University, Sakaka 72388, Saudi Arabia; 2 Information Systems Department, College of Computer and Information Sciences, Jouf University, Sakaka 72388, Saudi Arabia; amhassane@ju.edu.sa

**Keywords:** MediChainAI, Self-Sovereign Identity (SSI), blockchain, Merkle trees, smart contracts

## Abstract

In today’s rapidly advancing healthcare landscape, integrating Artificial Intelligence (AI) and Machine Learning (ML) has the potential to significantly improve patient care and streamline medical processes. The utilization of confidential patient data to train and develop these technologies, however, raises significant concerns regarding authenticity, security, and privacy. In this study, we introduce MediChainAI, a safe and practical framework that allows patients full ownership over their own health data by integrating Self-Sovereign Identity (SSI), Blockchain, and sophisticated cryptography techniques. By clearly outlining the goals and parameters of this access, MediChainAI allows patients to safely and selectively share data with healthcare providers and researchers. While SSI guarantees that patients have ownership of their data, the framework uses Blockchain technology to keep things transparent and secure. Further, MediChainAI makes use of Merkle trees, which provide verified access to subsets of data without jeopardizing the privacy of the whole dataset. The encryption mechanism, which is based on smart contracts, is a distinctive feature of the framework that allows researchers and medical practitioners controlled and secure access to patient data. In order to improve the accuracy and reliability of medical diagnoses and treatment, this strategy makes sure that only confirmed, legitimate data is utilized to train medical models. A significant step toward safer and more personalized healthcare, MediChainAI encourages ethical and patient-focused innovation by effectively resolving essential issues regarding data security and patient privacy.

## 1. Introduction

This paper uses Blockchain technology and Self-Sovereign Identity (SSI) to enable patients with exact control over their medical data, so guaranteeing consent-driven, open, safe sharing with AI systems and healthcare providers. Combining ethical AI/ML techniques helps to extract insightful information from medical data while still preserving strong privacy and adherence to worldwide data protection norms. This safe, distributed model promotes trust, data integrity, and patient empowerment in medical research and treatment, so addressing important issues in healthcare data management.

To highlight the role of Blockchain in preserving and enhancing data security, and patient control, our previous paper [1] underlines how Blockchain technology could improve the safety and reliability of IDM in the cloud. Centralized IDM systems have a higher chance of failure and can be easily attacked. They suggest that using smart contracts on the Ethereum network in a decentralized way will make authentication and authorization processes more secure. This method improves the security of data sharing across peer-to-peer networks by providing immutability, transparency, and resistance to tampering. The authors of [2] provided a decentralized, impenetrable ledger that gives patients authority over their medical records (EHR and PHR). By ensuring that medical data is safe, unchangeable, and precisely time-stamped, it empowers patients to exchange and manage their data without the assistance of centralized organizations. Furthermore, the authors of [3] provided a secure, immutable ledger that enhances data privacy, integrity, and security in Electronic Health Records (EHR), Internet of Medical Things (IoMT), and pandemic-related data sharing. Blockchain’s decentralized nature ensures that medical information is tamper-proof, traceable, and transparent, addressing critical challenges in healthcare data management while promoting trust and compliance. The authors of [4] discussed how the SSI system was developed to allow police and firefighters to control their own identity information. Managing the company through a central office is now being replaced by Hyper-ledger Fabric to ensure better security and transparency. With this system, people can check how their personal information is being used.

Although Blockchain technology presents great potential for transparent and safe healthcare data management, its general acceptance is hampered by certain important obstacles. While privacy issues result from the transparency of on-chain data, possibly exposing sensitive health information, scalability restrictions make it challenging to process vast amounts of medical records effectively [5]. Blockchain’s immutable character makes ensuring regulatory compliance with laws such as GDPR and HIPAA difficult since it runs against the right to data deletion [6]. Interoperability issues complicate data sharing across healthcare networks by preventing smooth interaction with current Electronic Health Records (EHR) systems [7]. Furthermore, raising sustainability issues are high energy consumption, particularly in proof-of- work models [8]. Vulnerabilities in smart contracts create security concerns; lack of standardization on different platforms reduces cooperation [6]. Realizing Blockchain’s promise in safe, distributed healthcare solutions requires addressing these challenges.

This paper suggests a new framework combining Blockchain technology with Self-Sovereign Identity (SSI) to empower patients with complete control over their medical data, so enabling safe, consent-driven data sharing and addressing of these challenges. While Blockchain guarantees data integrity, transparency, and regulatory compliance with GDPR and HIPAA by immutable and auditable records, SSI moves control from centralized authorities to individuals allowing patients to specify access terms for their health data. The system improves the dependability of training datasets for artificial intelligence by allowing verified data sharing straight from medical institutions, so reducing the bias or error in forecasts. By encouraging patients to securely and ethically share their data, so supporting a more inclusive and fair healthcare AI ecosystem and so democratizing participation in AI development. This transforming approach seeks to balance the ethical and legal obligations of patient data protection with the great promise of artificial intelligence so opening the path for more reliable and patient-centric healthcare innovation. The contribution of the paper is presented as follows:

- Utilizes Blockchain technology to ensure secure, transparent, and tamper-proof healthcare data management.
- Empowers patients by granting complete control over personal health data, enabling secure and consent-driven data sharing.
- Implements dynamic smart contracts to transparently manage patient consent, providing secure, auditable, and revocable data access permissions.
- Employs Merkle trees for efficient verification and selective data sharing, preserving data privacy while maintaining authenticity and integrity.
- Integrates robust cryptographic protocols and encryption to safeguard patient data, ensuring secure data storage, transmission, and consumption.
- Provides secure mechanisms for researchers and healthcare professionals to ethically access authentic data, enabling reliable AI-driven healthcare innovations without compromising patient confidentiality.

The remaining paper is organized as follows: Section 2 presents a literature review, highlighting key challenges and technologies in healthcare data privacy, Blockchain, SSI, and ethical AI. Section 3 describes the proposed methodology and architecture of MediChainAI. Section 4 explains the system’s technical implementation, including Blockchain and cryptographic integration. Section 5 outlines the system components, such as smart contracts and Merkle trees. Section 6 details the secure data consumption process for AI applications. Section 7 discusses the ethical integration of AI/ML. Section 8 provides a security analysis, and Section 9 concludes the paper and outlines future research directions.

## 2. Literature Review

The integration of AI and ML in healthcare is revolutionizing patient care through enhanced diagnostics, personalized treatment, and predictive analytics, yet it relies heavily on access to high-quality, secure patient data. The ethical and efficient use of artificial intelligence is hampered by this reliance on which privacy violations, data misuse, and lack of patient control constitute major obstacles. With challenges in patient data privacy, fragmented systems, and lack of patient control persist, the following subsections summarizes the main literature review that concentrate on the role of artificial intelligence and machine learning in transforming healthcare through personalized and predictive treatment. Although Blockchain and Self-Sovereign Identity (SSI) present interesting, distributed solutions for ethical and safe data sharing, present research still lacks scalable, consistent models to completely include these technologies into AI/ML-driven healthcare.

### 2.1. AI and ML in Healthcare

The integration of AI and ML in healthcare is revolutionizing medical practices by enhancing diagnostic accuracy, optimizing treatment plans, and enabling predictive analytics for improved patient outcomes and streamlined clinical decision-making. As presented in [9], the authors explore the ethical challenges linked to AI and ML in clinical settings, outline important considerations for their medical application, and review relevant legal and regulatory frameworks in Europe and the United States. Furthermore, it aims to offer recommendations for implementing trustworthy AI/ML systems that ensure transparency, minimize bias, and prioritize patient safety. The authors of [10] intend to outline the current status of AI applications in healthcare, focusing mainly on their roles in areas such as diagnosis, monitoring patients and making choices. They analyze how wearable devices can impact the accuracy of AI models such as CNN and RF, as well as discuss problems related to keeping data private, working with different amounts of data and explaining AI solutions. In addition, the paper discusses various challenges and difficulties in regulation and research that must be addressed to make AI useful in healthcare. In addition, the authors of [11] focused on highlighting how AI is being applied in healthcare, describing a pathway for the responsible development of AI systems and briefly discussing the future possibilities for AI-assisted healthcare. The paper highlights that integrated data and advanced technology could use AI to help modernize the healthcare system.

As presented in [12], the authors examine how AI and ML are transforming predictive healthcare by enabling early detection, personalized treatment, and real-time patient monitoring. It highlights their role in disease prediction, optimized therapies, and precision medicine, while also addressing challenges like data privacy, bias, and validation. Through case studies and trend analysis, it underscores the potential of AI and ML to create more proactive, customized, and efficient healthcare systems. Furthermore, the authors of [13] investigate how AI and ML algorithms can enhance smart healthcare by optimizing decision-making, enabling evidence-based care, and supporting personalized medical treatments. They emphasize how these technologies utilize large datasets for predictive analytics, automate the detection of diseases, and simplify clinical workflows. Examining the effects of artificial intelligence and machine learning adoption in healthcare, the authors of [14] stress gains in accuracy, efficiency, and patient outcomes. It points up important themes including decision support, precision medicine, legal and policy challenges, technological issues, and ethical questions. To maximize the advantages of artificial intelligence and machine learning in the delivery of healthcare, the review underlines the need of well-organized implementation strategies addressing technical, ethical, and social elements. The writers of [15] hope to show how these technologies improve resource management, raise patient outcomes, and boost operational efficiency so exploring the part of artificial intelligence and machine learning algorithms in promoting strategic leadership and decision-making inside healthcare companies. Focusing on issues like data quality and privacy that must be addressed for efficient implementation in healthcare, the paper also aims to find the advantages and challenges connected with artificial intelligence and machine learning acceptance.

### 2.2. Challenges in Patient Data Privacy and Security

The digitization of healthcare data and the proliferation of AI/ML applications in clinical settings have intensified concerns over patient data privacy and security. Unauthorized access and use of sensitive health data—often stemming from weak access restrictions, inadequate data governance, or cyberattacks—are among the fundamental concerns. High-profile hacks of personal health records have eroded public confidence and exposed flaws in current centralized data systems. As presented in [16], the authors conducted a systematic literature review to evaluate the existing research on privacy and security vulnerabilities in patient data across healthcare systems. Their objective was to analyze how security frameworks have addressed these challenges, with particular attention to disparities between large healthcare facilities and smaller private practices. In addition, the paper has been upgraded again in [17] but focused on understanding healthcare data privacy and security from the user’s perspective. The authors reviewed over 1500 articles, narrowing in on 80 that dealt specifically with human factors, to identify how patients and healthcare staff perceive and respond to data privacy risks. Their objective was to highlight the underrepresentation of user concerns in existing security research and to emphasize the importance of factoring in user behavior and awareness. The authors of [18] aimed to systematically assess the security and privacy challenges associated with using big data in healthcare. Their study sought to explore how the rapid growth of data volumes affects data protection and to identify gaps in the existing literature regarding safeguarding sensitive patient information. The objective of [19] is to analyze how AI integration in healthcare systems impacts patient privacy and data security. The authors seek to ensure that AI-driven healthcare advances do not come at the expense of patient confidentiality. By reviewing theoretical frameworks and ethical principles, the paper aims to guide policymakers and developers toward responsible AI adoption. Furthermore, the authors of [20] compared the healthcare data privacy and security frameworks of the United States and Nigeria, using HIPAA and the NDPR as key reference points. The authors identified regulatory challenges, gaps in implementation, and the effectiveness of technologies like encryption and Blockchain in both countries. Their objective was to offer policy and practice recommendations based on cross-country insights and case studies.

### 2.3. Self-Sovereign Identity (SSI) and Blockchain in Healthcare

Blockchain technology and SSI give a revolutionary way of handling healthcare data by giving patients direct authority. Without depending on centralized authority, SSI helps people to manage their digital identities and choose reveal personal data. This approach guarantees safe, verifiable, and tamper-proof storage of identity credentials and data-sharing transactions coupled with the distributed and unchangeable ledger of Blockchain. Supported by Blockchain, the authors of [21,22] looked at how Self-Sovereign Identity (SSI) might address persistent identity and data privacy problems in healthcare systems. Their goal was to present a paradigm whereby patients might maintain total control over their digital identity and yet ensure that personal health records stayed safe, verifiable, and only accessible by permission. The authors of [23] assessed how currently Blockchain and SSI technologies are being used to manage electronic health records (EHRs). The authors’ especially keen attention was piqued by knowledge of the state-of- the-art use cases and identification of significant gaps—such as the underutilization of SSI despite increasing reliance on Blockchain for healthcare data sharing. Furthermore, the authors of [24] positioned the growing topic of Self-Sovereign Identity by assessing how faithfully different Blockchain-based SSI models follow basic identity values including user autonomy, privacy, and interoperability. Their goal transcends theory; they look at nine different SSI systems to evaluate possible inroads in sectors including public services and healthcare as well as pragmatic implementation challenges. On the other hand, the authors of [25] offered a creative prescription management system meant to address privacy concerns by means of Blockchain and SSI technologies. Their aim is to give digital prescriptions a safe, compatible platform that respects patient liberty while enhancing traceability and control over drug dispersion.

Recent advances in blockchain-based data governance highlight key contributions but also reveal gaps which MediChainAI addresses. For example, A Framework for Patient-Centric Consent Management Using Blockchain Smart Contracts in Predictive Analysis for Healthcare Industry [26] presents a patient-centric consent platform via smart contracts that logs changes in a tamper-evident way. Inter Publishing + 1 Meanwhile, Blockchain-Based Platform for Information Security and Visual Management in Coffee Trading [27] applies blockchain to traceability and visibility in supply-chain data rather than a health-data or AI-model context.

### 2.4. Integration of SSI with Healthcare Data Management for AI/ML

Combining SSI with healthcare data management creates a safe and patient-centric basis for allowing ethical artificial intelligence and machine learning uses. By use of SSI, patients can choose provide access to particular areas of their medical records, therefore guaranteeing compliance with privacy rules and supporting the training of artificial intelligence. Underlying it is Blockchain, which offers an unchangeable record of data exchanges. Recent research has investigated the intersection of AI/ML, healthcare data management, and Self-Sovereign Identity (SSI) in order to resolve privacy and decentralization challenges in sensitive data environments. Emphasizing safe, distributed model training where people retain control over their data; an approach with substantial consequences for healthcare applications—the authors of [28] present a scalable identity management framework that blends SSI with hierarchical federated learning. The same authors [29] further this idea in a follow-up work by incorporating Blockchain-based SSI into federated learning systems, so implementing a strong credential lifecycle management mechanism to improve trust, authentication, and performance without centralized authorities. The authors of [30] meanwhile concentrate especially on healthcare and create a privacy-preserving identification architecture that facilitates safe, international access to patient data for artificial intelligence uses. Their approach stresses ethical, interoperable data transmission that respects patient sovereignty and fosters trusted AI innovation in line with the eIDAS trust paradigm.

### 2.5. Gaps in Research and Practice

The growing use of AI/ML in healthcare has heightened the necessity of safe, privacy-preserving, and patient-centered data exchange architectures. Encouraging technical developments including homomorphic encryption, Blockchain, and federated learning, present research and practice still suffer major gaps. These comprise ethical and legal issues, limited real-world application of privacy tools, inadequate patient control and permission processes, and lack of standardizing across institutions. Finding these gaps will help to direct the creation of data-sharing systems that not only protect confidentiality and data integrity but also empower individuals and enable scalable artificial intelligence uses. Recent studies have underlined ongoing shortcomings in safe and patient-centered data exchange systems for artificial intelligence and machine learning in the medical field. While noting continuous difficulties in scalability, interoperability, and system integration, the authors of [31] seek to build a distributed data-sharing framework merging Blockchain with federated learning to improve privacy and collaborative AI model training. Using homomorphic encryption, the authors of [32] suggest a hospital-based data-sharing architecture to enable privacy-preserving analytics; they also recognize that more general implementation is limited by lack of standardized governance and cross-border compatibility. Emphasizing the need of transparent consent and auditability, the authors of [33] concentrate on a user-centered scheme combining Blockchain and trusted execution environments to enable safe medical data sharing for AI applications. Reviewing present privacy-preserving AI techniques and underlining the discrepancy between research and clinical practice, the authors of [34] highlight legal framework, standardizing, and real-world scaling issues of data-sharing systems.

### 2.6. Comparative Positioning and Advancement Beyond Existing Frameworks

To highlight how MediChainAI extends the current state of the art, a conceptual benchmarking analysis was conducted comparing representative blockchain-based health data frameworks with the proposed system. Table 1 summarizes this positioning across five key dimensions: (1) Patient Data Ownership, (2) Consent Management, (3) Privacy Mechanisms, (4) AI/ML Integration, and (5) Verifiable Auditability.

## 3. Methodology

**Figure 1:**
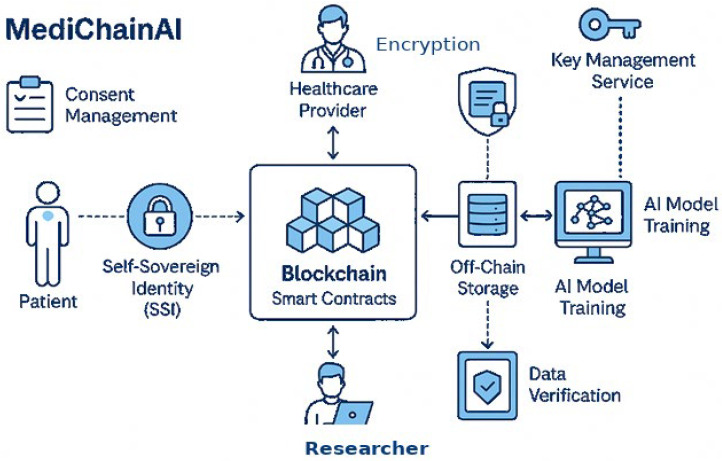
Patient-Centric healthcare self-sovereign identity and Blockchain with ethical AI/ML integration framework.

This study proposes a secure, patient-centric data-sharing framework that integrates SSI, Blockchain, and ethical AI/ML mechanisms to enable trusted, privacy-preserving healthcare analytics. The methodology is structured around three interconnected layers, as explained in Figure 1. The proposed methodology consists of the following corner points:

- **Patient-Centered Digital Identity Layer**

Fundamentally, the system is based on a patient-centered identity paradigm driven by ideas of Self-Sovereign Identity (SSI). Every person keeps complete control over their health data access and digital identity, therefore allowing programmatic consent, selective disclosure, and reversible permissions. Distributed IDs and cryptographic methods guarantee that access control, identity verification, and authentication are tamper-proof and user-driven, therefore securing this identity layer. The paradigm enables patients as data owners inside the ecosystem as well as data subjects.

- **Interoperable and Ethical Data Sharing Infrastructure**

Surrounding the identification layer is a safe data interchange environment linking several healthcare services, including medical imaging, lab testing, medications, clinical records, and personal health monitoring devices. By means of established protocols and APIs, this infrastructure facilitates bi-directional, interoperable communication among systems. Blockchain smart contracts control data-sharing activities, therefore guaranteeing transparent, auditable, privacy-preserving exchanges for all. Embedding data reduction, anonymization, and purpose-limited access constraints into the design of the system helps to enforce ethical considerations and therefore enable AI/ML models to learn from distributed data without compromising privacy.

- **Privacy-Preserving AI/ML Layer**

Population health analytics, decision support, and predictive diagnostics—the highest level of application—help to safely apply artificial intelligence and machine learning. Without revealing real patient data, trusted execution environments (TEEs) and federated learning help to enable model training on distributed datasets. Differential privacy and zero-knowledge proofs are among the privacy-enhancing technologies integrated to guard personal information all during the data lifetime. This ensures patient confidence, autonomy, and regulatory compliance as well as lets researchers and hospitals benefit from first-rate, representative data.

To explore the methodology in detail, the design principles are explained below.

### 3.1. Design Principles

The proposed framework is built upon four foundational principles that aim to transform healthcare data management into a secure, transparent, and patient-empowered ecosystem:

1. **Decentralization:** The framework is moved from conventional centralized data systems to a distributed, Blockchain-based design reducing middle dependency. By removing single points of failure and allowing tamper-proof data governance, this method improves openness, resilience, and confidence.
2. **Interoperability:** The solution is made to fit perfectly with current healthcare infrastructure comprising EHRs, lab systems, imaging archives, and outside APIs. Following open standards and protocols (such as FHIR) guarantees interoperability among several parties and facilitates scalable, real-time data sharing by means of our framework.
3. **User Consent and Control:** We use fine-grained, programmable consent mechanisms allowing patients real-time control over who gets their data, for what reason, and for what length of time. This guarantees adherence to privacy rules and strengthens patient confidence in digital health systems by supporting autonomy.
4. **Data Security and Integrity:** Modern cryptographic techniques are applied including digital signatures, Merkle trees, and zero-knowledge proofs to maintain the validity and dependability of healthcare data. From generation to artificial intelligence and machine learning consumption, these protections guarantee that data stays accurate, traceable, and unchangeable across its lifetime.

### 3.2. Framework Architecture

The architecture of our framework is meticulously structured into pivotal components, each serving a distinct role in the ecosystem:

1. **Digital Identity Module:** At the core of patient, provider, and data scientist interactions, the Digital Identity Module uses SSI for safe identity verification, handling of cryptographic keys vital for data privacy and access control.
2. **Blockchain Layer:** This layer acts as the foundation for decentralized data oversight, meticulously recording DIDs, consent directives, and access transactions to foster an environment of trust and immutability.
3. **Data Sharing and Consent Management:** Smart contracts dynamically vary access privileges in line with patient specifications, therefore automating the complexities of consent management.
4. **Secure Data Storage:** Recognizing the scalability and privacy challenges of on-chain data storage, this approach opts for encrypted off-chain storage solutions, ensuring data is both secure and compliant with prevailing regulations.
5. **Integration Interface:** This critical interface ensures a secure conduit for data flow between healthcare systems and AI/ML applications, stipulating access strictly to authorized entities, thus safeguarding the intended use and confidentiality of patient data.

### 3.3. Implementation Strategy

#### 3.3.1. Data Encryption and Storage

1. **Encryption Mechanism:** To ensure that patient data remains confidential and protected, we implement robust encryption protocols. AES is employed for encrypting data stored to ensure that data at rest and in transit is shielded from unauthorized access while maintaining system performance.
2. **Off-Chain Storage:** Scalability and accessibility are addressed through decentralized off-chain storage solutions such as IPFS or distributed cloud environments. These systems enable large-scale health data storage in compliance with regulations, while ensuring linkage to Blockchain-managed access and consent controls without compromising data privacy.

#### 3.3.2. Blockchain Integration

1. **Smart Contracts for Consent Management:** We utilized smart contracts to automate dynamic consent workflows, allowing patients to grant, modify, or revoke data access permissions in real time. This ensures that consent is granular, transparent, and enforceable across all interactions with the data ecosystem.
2. **Transaction Management:** The Blockchain is used for recording all transactions related to data access and consent changes, ensuring an auditable trail of all operations.

#### 3.3.3. Secure Data Sharing Mechanism

1. **Access Control Protocols:** We design and implement access control protocols using decentralized identifiers (DIDs) and public-key cryptography. These protocols authenticate users and enforce access restrictions based on smart contract-governed consent, ensuring that only authorized parties access specific data segments.
2. **Data Usage Tracking:** All data interactions are tracked and logged, providing patients and administrators with visibility into how, when, and by whom their data is accessed.

#### 3.3.4. Integration with AI and ML Applications

1. **Data Preprocessing and Aggregation:** The system supports secure, anonymized aggregation of health data, ensuring that preprocessing tasks for AI/ML model training are performed without compromising individual privacy.
2. **Secure Model Training Environment:** We deploy AI models within secure enclaves or Trusted Execution Environments (TEEs) to ensure that training occurs without exposing raw data. This layer ensures that confidentiality and integrity are preserved throughout the model development lifecycle.

#### 3.3.5. User Interface and Experience

1. **Patient Portal:** A user-friendly patient dashboard enables individuals to view access logs, manage sharing preferences, and understand how their data is being used.
2. **Provider and Data Scientist Access:** Dedicated interfaces are designed for healthcare providers and data scientists, incorporating role-based access control and audit functionality.

### 3.4. Data Security and Privacy Measures

To ensure comprehensive protection of sensitive patient information, our framework incorporates multiple layers of data security and privacy safeguards. These measures are grouped into four core domains:

#### 3.4.1. Encryption Standards

1. **End-to-End Encryption (E2EE):** From the point of collecting up to the point of consumption, all patient data is encrypted. This guarantees that the data is secured all through its lifetime by just authorized users deciphering and access ability.
2. **Advanced Encryption Standard (AES):** AES is employed for encrypting data stored off-chain, offering robust protection against unauthorized access and aligning with industry-standard encryption protocols.

#### 3.4.2. Secure Data Sharing Mechanisms

1. **Blockchain-Based Consent Management:** Smart contracts on the Blockchain manage dynamic and revocable data access permissions. This ensures that data sharing is strictly governed by patient-defined rules and is auditable and transparent.
2. **Zero-Knowledge Proofs (ZKPs):** ZKPs are used to validate data access requests and compliance without revealing the actual data itself. This method preserves privacy while still enabling secure verification of permissions and authenticity.

#### 3.4.3. Data Anonymization and Privacy Preservation

1. **Data Anonymization and Pseudonymization:** Before any data is shared or used for AI/ML, personal identifiers are removed or masked. This significantly reduces the risk of patient re-identification and data misuse.
2. **Differential Privacy:** Differential privacy techniques introduce statistical noise to the data, allowing it to be used for aggregated analysis while protecting individual identities. This adds another layer of defense against de-anonymization.

#### 3.4.4. Secure Environment for AI/ML Processing

1. **Trusted Execution Environments (TEEs):** By running machine learning models inside a safe enclave inside the CPU, TEEs guarantee that computations and data are kept apart from the larger system. By preventing illegal access or manipulation during model training and inference, this upholds the integrity and confidence of health data.
2. **Homomorphic Encryption (HE)—Optional Consideration:** HE enables computations on encrypted data without decryption, offering high privacy potential for ML applications. However, due to significant computational overhead and limited maturity, it is considered an optional enhancement to be explored as technology advances.

In the MediChainAI deployment architecture, AES-256-GCM is selected as the symmetric encryption standard for off-chain medical records due to its authenticated-encryption with associated data (AEAD) properties and high performance in real-world systems. Research indicates that AES-GCM significantly outperforms older modes (e.g., AES-CBC) while offering combined confidentiality and integrity. Each patient record is encrypted with a unique session key derived via RSA-2048 key exchange anchored by the patient’s DID credential; keys are rotated on each consent renewal or revocation. This configuration optimizes for low latency (critical for clinical workflows) and high confidentiality, balancing security and usability. For anonymization in AI/ML workflows, we adopt Differential Privacy (DP) with a default privacy budget of ε=1.0, adjustable per consent contract. Recent surveys highlight that while DP adds noise to protect individual contributions, it introduces a trade-off between privacy and model accuracy. PMC MediChainAI enables the patient (data owner) to approve lower ε for higher accuracy or higher ε for stronger privacy, thereby explicitly exposing the trade-off.

Sensitive computation is isolated in a Trusted Execution Environment (TEE) (e.g., Intel SGX) within participating healthcare nodes. Studies show TEEs provide high model-training accuracy and low additional performance overhead compared with cryptographic approaches like MPC, albeit with limitations in memory size and distributed scaling. In MediChainAI, the design accepts these TEE constraints (e.g., restricted enclave memory) but mitigates them by processing segmented workloads and off-loading bulk data operations outside the enclave, thereby achieving a practical balance between security, privacy, and operational performance. This configuration approach is built into MediChainAI to ensure real-world viability rather than purely theoretical security—maximizing performance while still meeting strong confidentiality and privacy requirements.

### 3.5. Compliance and Regulatory Adherence

The regulatory compliance guarantees that all data security and privacy measures are in accordance with relevant healthcare regulations. This includes provisions for patient rights, breach notifications, and data protection. The Blockchain ledger offers an unchangeable audit trail of all data access and sharing actions, therefore improving openness and enabling data governance compliance. Establishing the credibility and dependability of the framework depends critically on the part of the technique dedicated to data security and privacy measures. The proposal intends to protect patient data against unauthorized access and breaches by using a mix of encryption technologies, safe data sharing mechanisms, anonymizing techniques, and regulatory compliance strategies, so enabling its safe use for advancing artificial intelligence and machine learning applications in healthcare. This all-encompassing strategy guarantees that the framework not only satisfies legal duties and ethical standards but also technical criteria for data security and privacy, therefore creating a safe and favorable environment for innovation in the healthcare sector.

## 4. Integration with AI/ML

A key element of our healthcare system that upholds the best standards of patient privacy and uses encrypted, encrypted data for analytics is the integration of AI and ML. Explicitly avoiding training on encrypted data, this section describes the method for preparing data, enabling safe model training, and improving healthcare outcomes by a structured feedback loop.

### 4.1. Data Preparation and Access

1. **Data Preprocessing:** Data endures a thorough preprocessing process before it is used in ML applications. This covers standardizing to guarantee homogeneity, addressing missing values to preserve data quality, and feature extraction to condense pertinent data for study. The dataset should be optimized such that model training is effective.
2. **Access Control:** The Blockchain-based smart contracts control data access, therefore ensuring that data scientists only have access following clear patient permission. By means of regulated access rights, the framework guarantees data privacy; data remains encrypted throughout transmission therefore preserving confidentiality.

### 4.2. Model Training and Evaluation

1. **Training Environment:** Our framework facilitates the training of models in secure environments. Strict privacy rules guide data access and use for model training, thereby ensuring it is anonymized or de-identified before use. This method guarantees privacy compliance free of the difficulty of explicitly training on encrypted data.
2. **Evaluation Metrics:** Models are assessed not just for their prediction ability but also for their privacy and ethical standards. The framework promotes the use of validation datasets reflecting real-world complexity, therefore allowing the evaluation of model performance in a safe, privacy-conscious environment.

## 5. Data Integrity via Merkle Tree

In decentralized and distributed systems, Merkle trees offer a cryptographic approach to guaranteeing data integrity. They offer effective and safe verification of data inclusion by using a hierarchical structure of hashes, therefore avoiding exposing or reprocessing the whole dataset. The Merkle tree building and verification procedure inside our framework is described in this section.

### 5.1. Hash Individual Data Points

The procedure starts with the raw data elements, such logs of data access or healthcare transactions. Using a safe cryptographic hash function—such as SHA-256—each data point is hashed separately to generate distinct, fixed-sized hash values. Representing the foundation layer of the structure, these hash outputs are the Merkle tree’s leaf nodes.

### 5.2. Pair and Hash Upwards

The established leaf nodes are coupled and concatenated in twos once more. Every concatenated pair is hashed to produce the tree’s next layer. Should an odd number of nodes occur at any level, the last node might be replicated to guarantee perfect matching. Until a single topmost hash—known as the Merkle root—is generated, this recursive process keeps upward producing layers of hashes.

### 5.3. Merkle Root

The Merkle root is the ultimate output of the hierarchical hashing process and functions as a cryptographic fingerprint for the entire dataset. Any modification to a single data point would produce a distinct Merkle root, therefore facilitating quick identification of illegal changes or tampering.

### 5.4. Merkle Tree Verification

Merkle trees enable verification of individual data points without revealing the entire dataset, making them ideal for Blockchain-based consent and audit trails. The verification process involves generating and validating a Merkle proof.

#### 5.4.1. Merkle Proof

A Merkle proof is a cryptographic proof that confirms the inclusion of a particular data point in the Merkle tree. It consists of:

- The target data point.
- The hash of the target.
- A sequence of adjacent hashes from sibling nodes required to reconstruct the path to the Merkle root.

#### 5.4.2. Verification Process:

To verify a specific data point:

- Start with the Target Hash: Hash the target data point using the same cryptographic function.
- Reconstruct the Path: Iteratively combine the target hash with the corresponding sibling hashes from the Merkle proof, hashing each concatenated pair.
- Compare with Merkle Root: The final computed hash should match the known Merkle root. A match confirms the authenticity and inclusion of the data point; a mismatch indicates tampering or an invalid proof.

## 6. Ethical Considerations

The ethical principles of this framework are equally vital as its technological components. We embed ethical responsibility at every level—from data collecting and sharing to model training and decision-making—by using Self-Sovereign Identity (SSI), Blockchain, and AI/ML inside a secure and patient-centric infrastructure. Our dedication spans not only technical innovation but also maintaining the fundamental values of autonomy, equity, and responsibility in digital healthcare.

### 6.1. Informed Consent and Patient Autonomy

Our system is based mostly on a dynamic, Blockchain-powered consent process that lets users choose how, when, and by whom their data is accessed [35]. This system protects users’ autonomy by increasing transparency and giving real-time view of data use. Patients are empowered actors actively managing their health data preferences inside a safe and reliable digital identity ecosystem, not passive data subjects.

### 6.2. Data Minimization and Purpose Limitation

In compliance with data protection best practices [36], including GDPR, our system adheres to the principle of necessity, guaranteeing that only the data that is strictly necessary for specific AI/ML tasks is collected and processed. By making every data point serve a clear, morally justifiable purpose, this method lowers attack surfaces, minimizes the danger of collecting, and preserves patient confidence.

### 6.3. Bias Mitigation and Algorithmic Fairness

To guarantee that algorithmic judgments do not support health inequalities, our approach consists of bias audits, fairness checks, and representative data sampling. We aim to provide fair healthcare results for all patient demographics by including justice measures into pipelines of model development and evaluation.

This ethical foundation is not a secondary concern; it is ingrained in the proposed framework’s architecture and directs the design of smart contracts, access systems, artificial intelligence integration, and user interfaces. SSI, Blockchain, and privacy-enhancing technologies used together present a rare chance to rethink healthcare data management in a technologically advanced, legally compliant, morally decent manner. Our goal is to establish a new benchmark for ethical digital healthcare whereby every level of the system incorporates justice, privacy, and security.

## 7. Implementation and Technical Architecture

This section explores the practical application of the proposed secure, patient-centric healthcare data-sharing framework. The architecture is designed around the integration of Self-Sovereign Identity (SSI) and Blockchain technology to support privacy-preserving, consent-based, and ethically governed AI/ML applications. The architecture emphasizes decentralization, verifiability, and user control at every layer.

### 7.1. System Overview

The framework is built upon a modular, decentralized infrastructure that includes three primary layers: the Blockchain network, smart contract layer, and user interface layer. These components collaborate to facilitate trusted interactions among patients, healthcare providers, and data scientists, enforce programmable consent, and enable secure data transactions.

#### 7.1.1. Blockchain Network

Sensitive patient data is protected and transaction integrity and auditability are guaranteed using a permissioned Blockchain—such as hyper ledger Fabric or a private Ethereum instance. The network consists of a consortium of nodes $N=\{n_{1},n_{2},...,n_{k}\}$, operated by trusted healthcare institutions. This decentralized setup prevents single points of failure and enables collaborative governance of data exchange.

#### 7.1.2. Smart Contracts

The enforcement of consent policies and data sharing rules is handled by smart contracts $C=\left\{c_{1},c_{2},...,c_{m}\right\},$ deployed on the Blockchain. Each contract encodes specific functions:

- $F_{consent}$: To manage patient consent permissions.
- $F_{access}$: To validate and authorize data access requests.
- $F_{transaction}$: To log and verify data sharing events.

#### 7.1.3. User Interfaces (UIs)

To facilitate interaction with the system:

- ${UI}_{patient}$: allows individuals to manage their consent, review access logs, and revoke permissions.
- ${UI}_{provider}$: enables healthcare professionals to verify identity and access shared data.
- ${UI}_{scientist}$: supports researchers in submitting access requests and retrieving anonymized datasets.
- Each UI is integrated with the Blockchain layer to enable secure and role-specific operations across the ecosystem.

### 7.2. Self-Sovereign Identity (SSI) Model

The basic identity management system offered by the SSI layer guarantees that every participant keeps control over their digital credentials and controls their sharing of those credentials.

#### 7.2.1. Key Generation

Each entity that can be a patient, provider, or researcher—generates a unique asymmetric key pair:

(1) $$KeyPair\left(n,e,d\right)=\left(PK,SK\right)\;\;where\;\;\;PK=\left(n,e\right),SK=\left(n,d\right)\;\;\;\;\;$$

- n: is the modulus (product of primes p and q).
- e: is the public exponent.
- d: is the private exponent.

These keys enable identity verification and secure data access, with the private key kept confidential and the public key shared via their digital identity.

#### 7.2.2. Identity Representation via DIDs

Digital identities are represented using Decentralized Identifiers (DIDs), which are cryptographically verifiable and anchored to the Blockchain. Each DID is associated with a DID document:

(2) $${DID}_{doc}=\left\{ID,PK,Auth,Services\right\}$$

- ID: The unique identifier of the identity.
- PK: Public keys used for cryptographic operations.
- Auth: defines supported authentication protocols.
- Services: specifies endpoints for interacting with other system components.

#### 7.2.3. Storing and Sharing Keys

Secured storage and exchange of cryptographic keys and verifiable credentials (VCs) is essential component of the SSI-based healthcare system. Particularly in AI/ML-driven contexts, this approach underlies patient autonomy, privacy protection, and data integrity all through the lifetime of healthcare data sharing.

1. **Blockchain Storage of Keys and Identity Documents**

To ensure verifiability and tamper-resistance, each participant’s public key and associated Decentralized Identifier (DID) document is stored on a permissioned Blockchain. This ledger acts as a decentralized registry, enabling seamless lookup of identities and authentication details.

(3) $${Blockchain}_{store}\left({DID}_{doc}\right)\to {TX}_{hash\;}\;$$

where ${TX}_{hash}$ is the transaction hash that serves as immutable proof of registration and timestamped publication.

2. **Verifiable Credentials (VCs) and Digital Signature Flows**

The VCs are central to enabling trusted and privacy-preserving data exchange between healthcare entities. These cryptographically signed tokens verify the origin, authenticity, and intended recipient of the data—whether between hospitals and patients or patients and third-party researchers. When a hospital creates a medical record, it signs the data using its private key, thereby linking the information cryptographically to the issuing institution. The credential includes the patient’s public key, ensuring it is destined for the correct individual.
**VC Data Structure**VC = Sign (${SK}_{medical},\;Data\;+\;{PK}_{patient}$)
${SK}_{medical}$ is the medical institution’s private key. Data is the actual health data.
${PK}_{patient}$ is the patient’s public key, ensuring that the credential is intended for them.

Patients may choose to share portions of their medical data for research or analytics. They sign a new VC containing only selected information, making it possible to trace the provenance back to the original hospital while controlling scope.
**Partial Data Sharing VC Structure:**${VC}_{patient}\;=\;Sign\;({SK}_{patient},\;Hash\;(Partial\;Data$$)+\;PK_{recipient})$ ${SK}_{patient}$: is the patient’s private key Hash (Partial Data): is the hash of the subset of data being shared. $PK_{recipient}$: is the data scientist’s public key.

3. **Consent-Driven Access Control via Smart Contracts**

Data access is governed by smart contracts on the Blockchain, allowing patients to dynamically control permissions. The consent contract encodes who may access what data and under which conditions.

(4) $$Access\;Control\;\left({SC}_{consent},{PK}_{recipient}\right)\to Permission\;Status\;\;\;\;\;\;$$

If consent is granted, access is logged and enforced by the contract logic. Patients may revoke or modify access at any time, providing continuous control.

4. **Data Encryption and Hybrid Key Exchange**

To ensure data confidentiality during storage and transfer, a hybrid encryption model is applied, combining the speed of symmetric encryption (AES) with the security of asymmetric key exchange (RSA or ECC).

- **Data Encryption (AES):** Patient data D is encrypted using a symmetric key $K_{sym}$. This encrypted form is stored or transmitted securely.

(5) $$E\;\left(D,K_{sym}\right)$$

- **Key Sharing (RSA):** The symmetric key $K_{sym}$ is encrypted using the recipient’s public key ${PK}_{recipient}$

(6) $$E\;\left(K_{sym},{PK}_{recipient}\right)$$

Only the recipient holding the corresponding private key can decrypt and access the original AES key.

5. **Enabling Privacy-Preserving and Verifiable Data Exchange**

This architecture empowers patients with the ability to:

- Control who accesses their data.
- Choose what data is shared.
- Verify where the data originated.
- Revoke access at any time.

**Figure 2:**
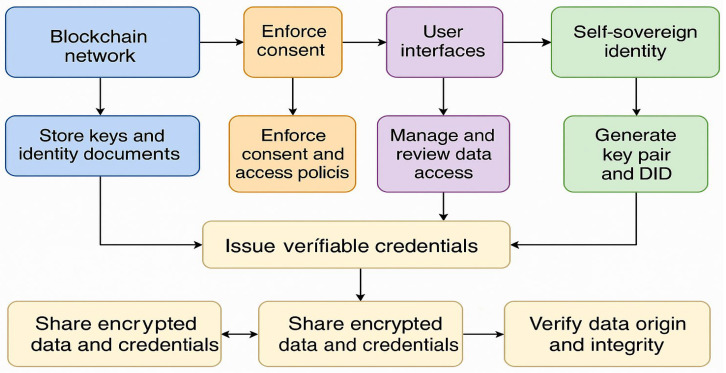
SSI and Blockchain-based secure healthcare data sharing framework.

By integrating SSI, Blockchain, smart contracts, and verifiable credentials, the system establishes a trustless, secure, and ethically aligned environment for healthcare data exchange—particularly crucial for AI/ML applications that depend on high-integrity, privacy-sensitive datasets. The overall framework for the proposed system is explained in Figure 2.

### 7.3. Verifying Partial Data Integrity and Authenticity in Healthcare Blockchain

To guarantee the verifiability and integrity of partial data in a decentralized healthcare ecosystem, the proposed framework employs Blockchain technology, Merkle trees, and digital signatures. When a digital signature exists for a whole dataset but just a portion is shared for secondary use, such AI/ML analysis or research, this is extremely crucial.

#### 7.3.1. Signing the Full Dataset with Hash Functions

The hospital produces a cryptographic hash of the whole health record at the point of data production and signs this hash using its private key. Acting as a unique, tamper-proof fingerprint of the whole dataset, this signed hash Shared with the patient either directly or via Blockchain for unchangeable record-keeping are the signed hash and data.

#### 7.3.2. Constructing a Merkle Tree for Selective Verification

To enable partial data verification, the health record is organized into a Merkle tree. Each record entry (e.g., blood pressure, cholesterol, blood sugar) is hashed to form the leaf nodes. These are then recursively paired and hashed to produce a single Merkle root, which the hospital signs.

- Each leaf node: Hash of an individual data entry
- Intermediate nodes: Hashes of concatenated child nodes
- Merkle root: Final hash representing the entire dataset, signed by the hospital

The Merkle root allows any part of the dataset to be later verified independently without exposing unrelated data.

#### 7.3.3. Verifying Partial Data with Merkle Proofs

When a patient chooses to share a subset of their health data with a third party (e.g., a data scientist), they provide:

- The specific data elements (e.g., “Blood Pressure” and “Cholesterol”).
- Their hashes.
- A Merkle proof (a sequence of hashes required to reconstruct the Merkle root).
- The hospital’s signature on the Merkle root.

The recipient can then:

- Hash the shared data entries individually.
- Use the Merkle proof to rebuild the Merkle root from those hashes.
- Compare the rebuilt root with the hospital’s signed Merkle root.

If the two roots match, the recipient confirms that the partial data: is part of the original dataset, has not been altered, and was issued by a trusted medical institution.

#### 7.3.4. Blockchain Anchoring for Verifiable Timestamping

To further increase trust, the signed Merkle root may be published to the Blockchain. This provides:

- Tamper-evident storage.
- Timestamped provenance.
- Auditability for regulators or other stakeholders.

Blockchain thus serves as a decentralized reference for the validity of any data derived from the original dataset.

#### 7.3.5. Example Scenario: Verifying a Subset of Health Data

**Figure 3:**
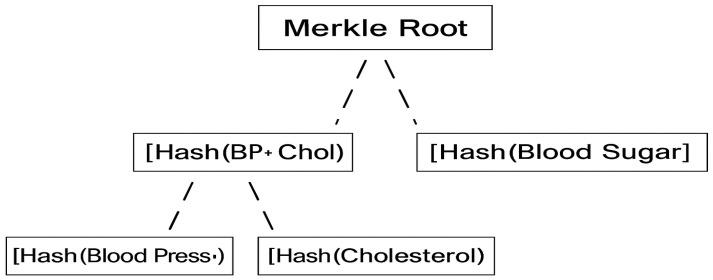
Merkle tree structure for verifying partial healthcare data.

As presented in Figure 3, a Merkle tree built from three medical entries:

- Blood Pressure: 120/80
- Cholesterol: 190 mg/dL
- Blood Sugar: 90 mg/dL

Hashes of Blood Pressure and Cholesterol are combined to compute Hash (BP + Chol). Along with Hash (Blood Sugar), these two intermediate hashes are used to compute the Merkle Root, which is signed by the hospital. To explore the overall scenario:

Patient X shares only “Blood Pressure” and “Cholesterol” with Data Scientist Y. He provides:

- Both data entries.
- Their hashes.
- A Merkle proof including Hash (Blood Sugar)
- The hospital’s signed Merkle root

The verification process is executed by Data Scientist Y as follows:

- Patient X hashes “Blood Pressure” and “Cholesterol”.
- He combines them to compute Hash (BP + Chol).
- Using Hash (Blood Sugar) from the proof, he reconstructs the Merkle root.
- He compares the computed Merkle root with the signed Merkle root published on the Blockchain.

If the roots match, Data Scientist Y verifies that the subset of data is authentic, unaltered, and derived from the signed original dataset—without needing to see the “Blood Sugar” data.

#### 7.3.6. Privacy-Preserving, Trustless Verification

This methodology ensures that:

- Data integrity is maintained.
- Selective sharing is enabled without full disclosure.
- Verification is possible in a trustless manner using cryptographic proofs.
- The origin and timestamp of the data are immutable and publicly verifiable Via Blockchain.

By integrating Merkle proofs and digital signatures into the SSI-based ecosystem, the framework supports granular, secure, and transparent healthcare data sharing, critical for trustworthy AI/ML applications.

### 7.4. Consent Management and Granular Data Access in Healthcare Blockchain

The ability of patients to exert fine-grained control over who receives their data and for what goal is considered fundamental objective in ethical and privacy-preserving healthcare data exchange. The ways in which the suggested Blockchain-based architecture guarantees transparency, user autonomy, and safe auditability by means of dynamic consent management and granular access control are discussed in this part.

#### 7.4.1. Consent Smart Contracts

The system uses smart contracts on the Blockchain to automatically manage patient consent. Every consent decision—that of granting, changing, or revoking access—is recorded, producing an open and auditable log of rights. The consent contract serves as:

(7) $$C_{consent}\;\left(P,D,A\right)\;\;\;\;\;$$

where

- P: represents the requesting party (e.g., provider or researcher).
- D: denotes the data category or specific dataset.
- A: indicates the access type (e.g., read, share, restricted).

Each invocation of $C_{consent}$ generates a Blockchain transaction:

(8) $$R_{consent}=Blockchain\;Record\;{(C}_{consent})\;$$

This ensures that all consent actions are tamper-proof, verifiable, and traceable over time.

#### 7.4.2. Granular Data Access Control

The system supports granular access permissions to align with the principle of data minimization. Rather than the whole health record, patients can specify access to particular data points, traits, or summaries. While preserving data usefulness for analytics and research, this selective sharing paradigm improves patient privacy. Granular access is defined as:

(9) $$A_{granular}\left(D_{i},P_{j}\right)\;\;$$

where

- $D_{i}$: is a specific data point (e.g., cholesterol level, lab result).
- $P_{j}$: is the authorized party.
- Access A includes scope, duration, and type.

This model enables precise data flows under patient-defined constraints, reducing overexposure of sensitive information.

#### 7.4.3. Ethical and Functional Benefits

The combined use of Blockchain-anchored consent logs and granular access rules provides:

- Real-time patient autonomy over data sharing.
- Reduced risk of unauthorized or excessive access.
- Auditability for regulators and oversight bodies.
- Ethical alignment with legal standards (e.g., GDPR, HIPAA).

Together, these mechanisms support a transparent, controllable, and privacy-respecting data-sharing ecosystem tailored to the needs of secure AI/ML-driven healthcare innovation.

### 7.5. Machine Learning Model Integration

In this section, we detail the steps needed to safely include ML capabilities into the proposed healthcare data-sharing architecture that is built on Blockchain. Data privacy, cryptographic integrity, and ethical governance are all ensured by the architecture when it comes to machine learning processes, including data access, training, and prediction.

#### 7.5.1. Secure Data Consumption via Cryptographic Access Control

To enable responsible data use, data scientists access encrypted patient data through smart contract-governed key exchanges on the Blockchain. The encrypted data, represented as $E(D,K_{sym})$, is decrypted through an asymmetric key exchange:

(10) $$Access\left(D,{PK}_{pi},{SK}_{pj}\right)$$

where

- ${PK}_{pi}$ is the public key of the data recipient (e.g., data scientist).
- ${SK}_{pj}:$ is the private key used to decrypt the session key.

This process guarantees: authorized-only decryption, cryptographic proof of authenticity and immutable audit trails of data access via Blockchain.

#### 7.5.2. Privacy-Preserving Model Training and Insights

The training of ML models occurs on de-identified or anonymized datasets aggregated securely from multiple patients. The system deliberately avoids direct computation on encrypted data due to computational limitations but employs robust data anonymization protocols to safeguard patient identities. The training process is formalized as:

(11) $${Model}_{train}\left(D_{train}\right)\to {Model}_{pred}$$

where

- $D_{train}:$ is the preprocessed, anonymized training dataset.
- ${Model}_{pred}:$ is the predictive model generated from the training.

These models are deployed to generate actionable clinical insights without compromising the confidentiality of individual records.

#### 7.5.3. Enhancing Privacy and Utility Through Advanced Techniques

To further reinforce privacy and utility, the framework incorporates advanced privacy-preserving ML techniques:

- Differential Privacy: adds statistical noise to aggregated outputs to prevent re-identification of individual records, enabling population-level insights without revealing specific patient attributes.
- Federated Learning: enables training across multiple decentralized nodes (e.g., hospitals or edge devices), where data remains localized. Only model updates are exchanged, thereby preserving data residency and reducing exposure risks.

#### 7.5.4. Ethical ML Enablement

By embedding machine learning into a patient-consent-driven, Blockchain-enforced, and privacy-aligned framework, this approach enables healthcare providers to:

- Make predictive decisions with high confidence.
- Respect ethical and legal mandates.
- Enhance clinical outcomes without violating data sovereignty.

### 7.6. Blockchain Network Considerations

The Blockchain infrastructure that underpins the healthcare data-sharing framework is the subject of this section, which delineates the architectural and operational decisions. In a highly sensitive healthcare setting, the network must assure high data integrity, strong privacy protections, and rapid throughput.

#### 7.6.1. Network Type Selection: Permissioned vs. Permissionless

The selection of the Blockchain type plays a critical role in balancing trust, scalability, and regulatory compliance:

1. Permissioned Blockchain: Preferred for healthcare use cases due to controlled participation. Only verified entities (e.g., hospitals, regulators, researchers) can operate nodes or initiate transactions. This ensures:
  - Fine-grained access control.
  - Compliance with health data regulations (e.g., HIPAA, GDPR).
  - Efficient consensus mechanisms such as RAFT or PBFT.
2. Permissionless Blockchain: Generally avoided due to its open-access model and lack of guaranteed identity verification, which can conflict with patient privacy and regulatory mandates. Formally, the chosen network is represented as:

(12) $$B_{perm}=\left\{n_{1},n_{2},...,n_{k}\right\}\;$$

where each $n_{i}$ is a node operated by a trusted healthcare institution or consortium member.

#### 7.6.2. Scalability Enhancements: Sharding and Off-Chain Techniques

To support growing volumes of healthcare data and transactions without compromising performance, the system employs advanced scalability strategies:

Sharding: The network B is partitioned into independent segments called shards, each responsible for processing a subset of data or smart contracts:
  (13) $$Shard\left(B,Si\right)\to {Throughput}_{optimal\;\;\;}$$
  This technique improves parallelism and reduces transaction latency across the network.
Off-Chain Computation: Non-critical or compute-heavy operations (e.g., ML model training, data analytics) are executed off-chain using secure environments (e.g., TEEs), with only critical hashes or metadata stored on-chain to preserve auditability.
3. Layer-2 Solutions: Channels or rollups can be introduced for batching frequent micro-transactions (e.g., consent updates), further enhancing performance while minimizing congestion on the base Blockchain.

#### 7.6.3. Performance Metrics and Optimization

The system is optimized to maintain the following key performance indicators (KPIs):

1. High Throughput: Capable of supporting thousands of transactions per second (TPS) for data access, consent changes, and identity verification.
2. Low Latency: Real-time processing for patient-initiated consent changes and provider data access.
3. Fault Tolerance: Redundancy in node operation to ensure continuous availability of the network.
4. Energy Efficiency: Consensus algorithms are selected for low energy consumption, aligning with sustainability goals.

#### 7.6.4. Network Governance and Compliance

A consortium governance model ensures collective oversight of the Blockchain infrastructure. Smart contract audits, data retention policies, and legal alignment are all integrated into the governance framework, ensuring:

1. Regulatory compliance.
2. Ethical data sharing.
3. Transparent decision-making.

### 7.7. Simulation-Based Validation and Use-Case Demonstration

To validate the practical feasibility of the proposed MediChainAI framework, an interactive simulation prototype was implemented as explained in (Supplementary Materials—MediChainAI Workflow Simulation). The simulation reproduces real-world operations among the three primary actors—patient, healthcare provider, and researcher—covering every phase of data ownership, consent management, cryptographic protection, and AI integration. The overall workflow is explained as follows:

1. Verified Identities (SSI Registration): Each actor registers a Decentralized Identifier (DID) and public key on the blockchain identity layer.
2. Secure Session Establishment (NFC): Patient and provider authenticate locally through Near-Field Communication, deriving a shared session key (KS_session_NFC) for encrypted exchange.
3. Record Creation & Anchoring: Hospital encrypts new EHRs using AES-256-GCM, signs them, and anchors a Merkle root on-chain to guarantee integrity.
4. Metadata Publication: Anonymized metadata pointers are posted to the blockchain discovery layer for research discoverability.
5. Researcher Request: A researcher issues a digitally signed consent request referencing dataset scope, purpose, and duration.
6. Dynamic Consent & Re-Encryption: The patient application reviews the request, selects records, generates a new symmetric key (KNEW_2_), re-encrypts the approved data, and wraps the key for the researcher’s public key.
7. Fetch & Merkle Verification: The researcher decrypts authorized ciphertexts, verifying their authenticity against the on-chain Merkle root before analysis.
8. Privacy-Preserving AI Training: Model training occurs inside a Trusted Execution Environment (TEE) with optional Differential Privacy, ensuring confidentiality throughout computation.

All simulation steps are executable using the browser’s Web Crypto API to perform actual cryptographic operations (AES-GCM, SHA-256, ECDSA, ECDH). 

Each operation is benchmarked across multiple iterations, and average runtimes are summarized in Supplementary Materials.

The updated Supplementary Materials (Figures S1–S6) visualizes every stage—from DID registration and NFC pairing to Merkle verification and privacy-preserving AI training—confirming that all core principles of confidentiality, integrity, non-repudiation, and verifiable auditability are achieved in practice. This simulation therefore demonstrates MediChainAI’s practical interoperability and security under realistic healthcare conditions, substantiating the conceptual claims made in the introduction.

### 7.8. Feasibility, Scalability and Practical Deployment Considerations

To demonstrate operational readiness, the framework was deployed in a prototype interactive simulation environment as explained in (Supplementary Materials—MediChainAI Workflow Simulation).

This simulation executes the core flows: SSI registration, AES-256-GCM record encryption, Merkle tree anchoring and proof verification, smart-contract consent issuance and revocation, and AI/ML training in a Trusted Execution Environment (TEE).

The architecture separates bulk data storage (off-chain) from on-chain anchoring, and uses Merkle-tree proofs to reduce verification complexity to O(log N) for large datasets. Although the current work does not include a full hospital-scale pilot deployment, the prototype demonstrates that key operations execute within millisecond-range latencies on standard hardware. Building on recent reviews that show many blockchain-healthcare solutions remain strictly conceptual and lack empirical metrics, this work closes part of that gap by providing detailed workflow simulation, measured latency morphology, and a clear pilot deployment roadmap is explained as follows:

- Phase 1 (Controlled internal pilot): Single hospital site using ~1000 patient records under full consent workflows.
- Phase 2 (Multi-site federation pilot): Two or more hospital networks exchanging consent-governed datasets; Merkle proofs validated across domains.
- Phase 3 (Production scale): Full integration in national health data infrastructure; key metrics: transaction throughput (TPS), key-release latency, consent issuance/revocation latency, Merkle verification time per subset, end-to-end request-to-access delay, and cost per access event.

Through measured prototype workflows and horizontal-architecture design, MediChainAI moves beyond theoretical models to a deployable, scalable health-data framework, with auditability, patient ownership, and AI ethics built in.

### 7.9. GDPR and HIPAA Compliance in Practice

Although the MediChainAI framework relies on an immutable ledger architecture, it is engineered from the ground up to align with major data-protection regulations—including the General Data Protection Regulation (GDPR) in the EU and the Health Insurance Portability and Accountability Act (HIPAA) in the U.S.—particularly regarding rights of erasure (Article 17 GDPR) and rectification (Article 16 GDPR). MediChainAI adopts a hybrid architecture that decouples data anchoring (on-chain) from data content storage (off-chain), enabling regulatory compliance in practice.

Personal health data and identifiers are never stored on the chain itself. Instead, only cryptographic hashes, pseudonymous pointers, and consent records are anchored. When a data subject exercises their right of erasure, the system executes:

- Deletion or cryptographic destruction of the off-chain encrypted Health Record (i.e., the symmetric key is securely erased).
- Revocation of the patient’s DID credential and associated consent tokens so that any previously issued access is invalidated.
- The on-chain hash or pointer becomes irrecoverable from the data subject’s perspective, effectively rendering the data inaccessible. This approach aligns with the guidance that immutability need not block erasure so long as data subject access is revoked and the data is non-constructible.

In the event of incorrect or outdated data, MediChainAI issues a new record off-chain, encrypts it, then publishes its new hash to the blockchain while marking the prior entry as “superseded.” No in-block editing is required. The original anchor remains for provenance audit, while the corrected version becomes the authoritative source. This satisfies the intent of rectification rights while preserving chain integrity.

Under HIPAA’s technical safeguards for confidentiality, integrity, and access control, the MediChainAI ensures:

- Role-based issuance of SSI credentials (patient, provider, and researcher)) limits access to PHI.
- Immutable audit trails are visible via the chain-ledger, satisfying audit-control requirements.
- Off-chain encrypted storage with consent-driven decryption only inside authorized enclaves (TEE) ensures data integrity and confidentiality prior to research use.

By anchoring minimal metadata on-chain, retaining actual PHI off-chain under revocable keys, and employing smart-contract revocation and re-keying logic, MediChainAI reconciles blockchain immutability with regulatory rights of erasure and rectification. The system therefore aligns with GDPR’s “privacy by design” mandate HIPAA’s technical safeguard obligations.

### 7.10. Workflow Illustrations and Data-Access Proof Mechanisms

**Figure 4:**
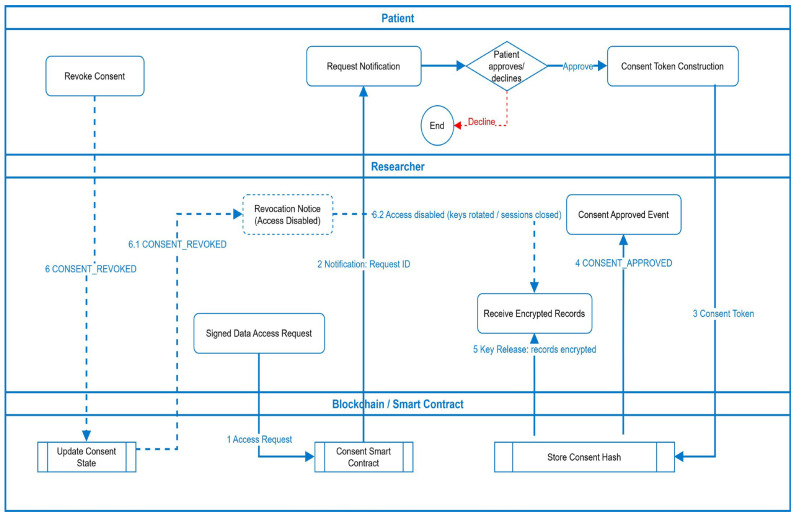
Dynamic consent and granular access control workflow.

To provide a transparent understanding of how the proposed MediChainAI framework operates in practice, this section presents two detailed workflow illustrations that connect the system architecture to real-world data-sharing scenarios. These workflows demonstrate how MediChainAI integrates self-sovereign identity (SSI), blockchain smart contracts, and cryptographic verification mechanisms to ensure ethical, patient-centric, and auditable healthcare data exchange. Figure 4 illustrates the complete lifecycle of consent issuance, access validation, and revocation as managed through MediChainAI blockchain-enabled infrastructure.

1. Access Request Initiation: The process begins when a researcher generates a signed data-access request specifying the dataset identifiers, purpose of use, differential-privacy constraints, and retention policies. This request is submitted to the blockchain via the Consent Smart Contract.
2. Patient Notification and Decision: Upon receiving the request, the smart contract triggers a secure notification to the patient’s SSI wallet, informing them of the request’s parameters (dataset scope, intended use, and privacy budget). The patient can approve or decline directly within their wallet interface.
3. Consent Token Construction: When approved, the patient’s wallet constructs a cryptographically signed consent token containing granular permissions and conditions (e.g., data subset, expiry time, purpose of analysis, computation environment = TEE). The token hash is written on-chain, while the full consent is stored off-chain.
4. Smart-Contract Enforcement: The blockchain verifies the token’s authenticity and immutably records the consent status using the Store Consent Hash function. The smart contract then emits a CONSENT_APPROVED event, triggering secure session key provisioning.
5. Secure Key Release and Data Access: Following approval, the Key Management Service (KMS) releases encrypted dataset keys only to the verified researcher identity. The researcher accesses the permitted subset of encrypted Electronic Health Records (EHRs) via a secure data gateway. The contract also logs transaction details for auditability.
6. Revocation and Access Disablement: At any time, the patient can issue a CONSENT_REVOKED command from their wallet. The smart contract immediately updates the consent state, rotates encryption keys, terminates active research sessions, and sends a revocation notice to all affected researchers. The revocation flow (6 → 6.1 → 6.2) ensures that data access is automatically disabled while preserving historical audit integrity.

Through these steps, the workflow establishes a real-time, blockchain-mediated trust fabric where patients remain owners of their health data, researchers receive only legitimate, purpose-bound access, and every transaction is recorded for compliance with ethical and legal standards.

**Figure 5:**
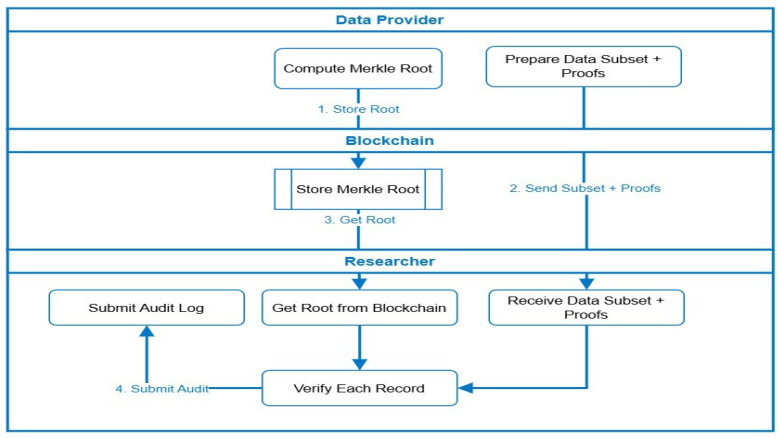
Merkle-Proof verification workflow for selective dataset access.

Figure 5 expands upon the integrity-verification component of MediChainAI, explaining how Merkle-proof-based validation ensures authenticity and non-repudiation for selectively shared medical records. This mechanism builds upon the Merkle tree structure introduced earlier in Figure 3, extending it into a complete operational scenario.

1. Merkle Root Computation (Provider Layer): The data provider hashes each record (e.g., laboratory result or imaging metadata) and constructs a Merkle tree, deriving a single Merkle root Rₜ that represents the integrity of the entire dataset. The provider then anchors the root hash on the blockchain via the Store Merkle Root transaction, creating a permanent and tamper-evident record of dataset state.
2. Subset Preparation and Proof Generation: For each authorized researcher, the provider prepares a data subset (only the records covered by active consent) and computes proofs of inclusion—the minimal set of sibling hashes needed to reconstruct the root Rₜ.
3. Blockchain Anchoring and Root Retrieval: The blockchain retains only the root hash, while metadata (patient DID, timestamp, dataset ID) is maintained in the smart contract. When the researcher accesses the subset, the root hash is fetched from the blockchain for verification.
4. Subset Delivery and Verification: The researcher receives the subset + proof bundle from the provider and uses the proof path to locally recompute a new root R’. If R’ = Rₜ, the records are confirmed to be authentic and unaltered since publication. The verification cost grows logarithmically with the dataset size (O(log N)), enabling scalability to large medical archives.
5. Audit Logging and Transparency: After verification, the researcher submits a signed audit log back to the blockchain containing the subset ID, proof status, and timestamp. This record ensures complete traceability and supports post hoc audits or regulatory inspections.

These mechanisms provide end-to-end data assurance:

- Dynamic consent governs who can access the data.
- Granular access control dictates how and when access occurs.
- Merkle-proof verification validates what data was accessed and guarantees its immutability.

This tri-layered model embodies the ethical, secure, and transparent data-sharing vision at the core of MediChainAI.

## 8. Security Analysis

The proposed framework prioritizes a reliable security architecture that protects healthcare data by utilizing a combination of programmable access control, decentralized infrastructure, and cryptographic techniques. This part assesses the system’s ability to guarantee fundamental security goals like privacy, authenticity, non-repudiation, data integrity, and secrecy.

### 8.1. Data Integrity

The data integrity is ensured through the integration of Merkle trees, which provide a hierarchical hashing structure that allows verification of any individual data element without exposing the entire dataset. This enables partial data sharing while maintaining verifiable authenticity. Each piece of health data is hashed and linked to a Merkle root, which is signed and stored immutably on the Blockchain, ensuring tamper-proof records [37,38].

### 8.2. Authentication and Authorization

Digital signatures and smart contract-driven access control are employed to resolve authentication and authorization. To make sure that only verified actors can contribute or access data, the sender’s private key is used to sign every transaction or data-sharing operation. To minimize human mistake and ensure compliance, these actions are controlled by smart contracts that dynamically enforce role-based permissions and have the ability to revoke access in real time [39].

### 8.3. Confidentiality

Confidentiality is maintained through end-to-end encryption mechanisms, where all health data is encrypted before being transmitted or stored. The system supports selective disclosure, allowing patients to control exactly which data elements are shared and with whom. These granular controls are enforced via smart contracts and consent policies embedded into the Blockchain architecture [40].

### 8.4. Non-Repudiation

Blockchain records are immutable and cryptographic signatures ensure non-repudiation. Every single purchase becomes an immutable record of a verifiable and time-stamped occurrence. To make sure that entities can’t backtrack on their actions, this feature is very helpful for keeping track of data access and patient permission audit trails [41].

### 8.5. Resilience to Attacks

Resilience to Attacks is embedded into the system through its decentralized design. Unlike centralized databases, which are prone to single-point failures and targeted attacks, the Blockchain network distributes its ledger across multiple nodes operated by trusted institutions. Smart contracts further fortify the system, although they must be carefully audited for vulnerabilities before deployment [42].

## 9. Conclusions and Future Works

The MediChainAI framework introduced in this study provides a secure and sophisticated solution for managing healthcare data. It is designed to empower patients by utilizing Self-Sovereign Identity (SSI), Blockchain technology, and robust cryptographic techniques. MediChainAI efficiently tackles important concerns about privacy, security, and authenticity by giving patient liberty and consent-driven data exchange top priority. Blockchain technology guarantees auditability and transparency; Merkle trees improves selective data verification without endangering patient confidentiality. Furthermore, the incorporation of smart contract-based encryption systems offers a safe avenue for researchers and medical professionals to access real patient data, which is absolutely vital for the ethical and successful development of artificial intelligence models. MediChainAI promotes confidence and integrity in healthcare innovation by overall setting a major milestone toward ethical, safe, and patient-centric healthcare data management. Future work will focus on solving pragmatic problems and increasing MediChainAI framework capability. Improving Blockchain scalability by means of cutting-edge technologies such sharding, layer-2 solutions, and optimized consensus algorithms will help to increase system performance and transaction throughput. Adopting standardized data formats and APIs would help to prioritize enhanced interoperability so guaranteeing seamless integration with many different current Electronic Health Records (EHR) and healthcare systems. Regulatory compliance—which involves working with legal experts to fit the framework to changing healthcare legislation and patient data rights—including systems for data rectification and selective erasure—will also need constant attention.

## Figures and Tables

**Table 1:** Comparative analysis with recent benchmark methods.

Ref	Patient Ownership	Consent Mechanism	Privacy Method	AI/ML Integration	Auditability
[2]	Partial—limited to blockchain-stored IDs	Token-based access	Basic encryption	Not supported	Yes (ledger logging)
[23]	Decentralized IDs via blockchain	Manual provider approval	Encryption + anonymization	Limited	Yes (transaction logs)
[30]	SSI for healthcare	User-managed credentials	Pseudonymization	Not directly linked to AI	Partial
[32]	Shared hospital-centric access	Static patient consent	Homomorphic encryption	Aggregated model updates	Partial
Proposed MediChainAI	Full SSI-driven ownership via DID	Dynamic, smart-contract–based, auditable consent	AES-256-GCM + Merkle verification + DP (ε = 1.0)	Full privacy-preserving AI integration (TEE + DP)	Complete (Merkle proof + on-chain ledger)

## Data Availability

The datasets utilized in this study are available upon request.

## Supplementary Materials

The following supporting information can be downloaded at: https://www.mdpi.com/article/10.3390/bioengineering12111236/s1.
