# Supplementary material for: Ethical AI in Healthcare: Integrating Zero-Knowledge Proofs and Smart Contracts for Transparent Data Governance
Source: Bioengineering (Basel). 2025 Nov 12;12(11):1236. doi: 10.3390/bioengineering12111236 (PMC12650700; doi:10.3390/bioengineering12111236)
Supplement: Supplementary file 1 [file bioengineering-12-01236-s001.zip › bioengineering-3926034-supplementary.pdf]

Supplementary Material

- **Actors:**

➢ *John Doe* (patient)
➢ *City General Hospital* (healthcare provider)
➢ *Dr. Sarah Chen* (researcher)

1) Step 1: Patient Identity Setup (SSI)

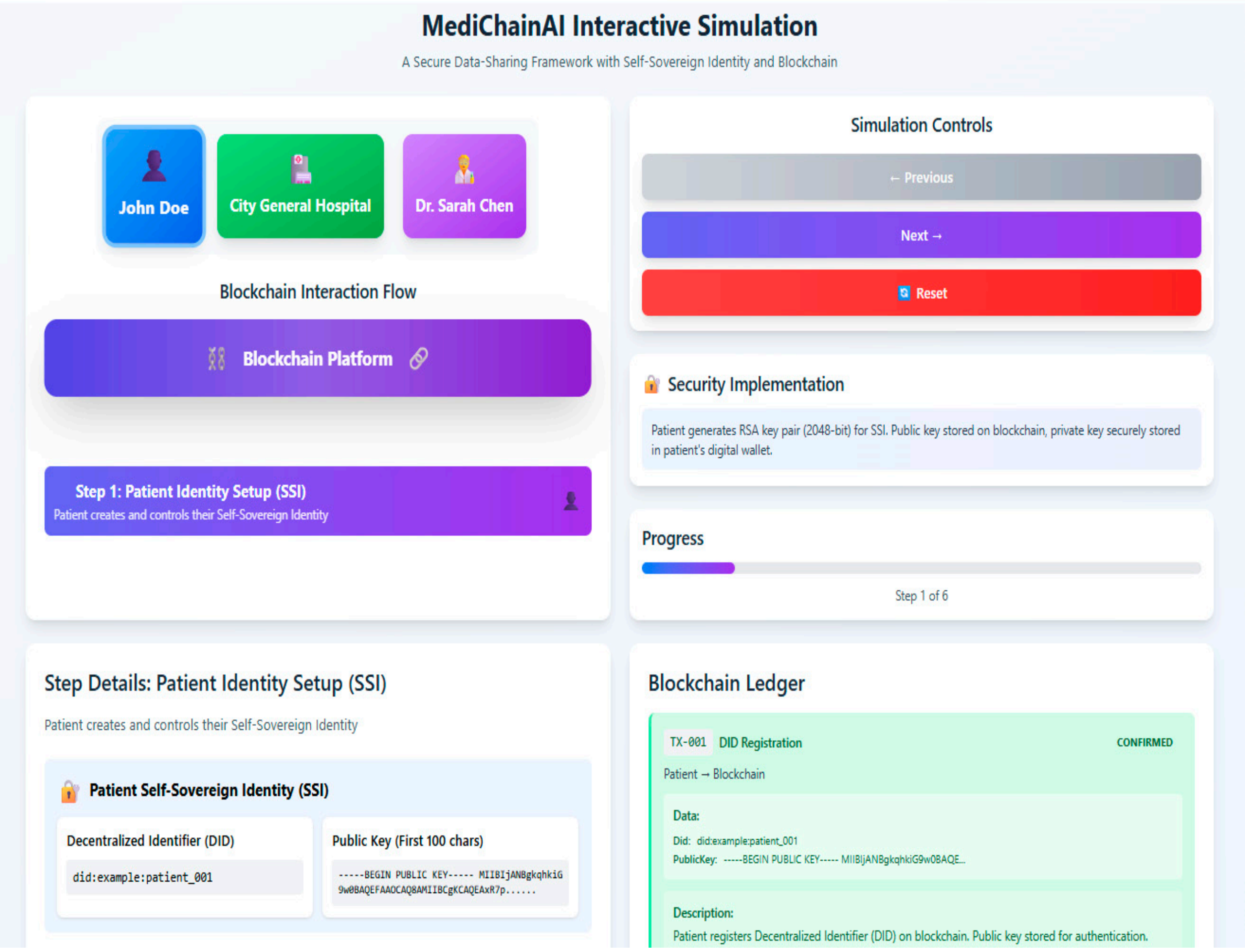

Figure S1: Patient Identity Setup (SSI)

The first step in the MediChainAI framework is the creation and registration of a Self-Sovereign Identity (SSI) for the patient.

- **Process flow:**
  - *The patient generates an RSA key pair (2048-bit) for identity.*
  - *The public key is stored securely on the blockchain ledger, ensuring immutability and verifiability.*
  - *The private key remains safely in the patient's digital wallet, under their full control.*
- **Interface features:**

- *The Blockchain Interaction Flow highlights that data exchange passes through the Blockchain layer.*
  - *The Simulation Controls allow navigation (Next, Previous, Reset).*
  - *Security Implementation clearly states the cryptographic process at this step.*
- **Blockchain Ledger Output:**
  - *Displays a Transaction ID (TX-001) for DID Registration.*
  - *Shows the registered Decentralized Identifier (DID) (e.g., did: example: patient_001).*
  - *Confirms that the public key is anchored to the Blockchain for authentication.*
- **Significance:**
  This step establishes the foundation of trust and identity in MediChainAI. By linking, the patient's DID and public key to the Blockchain, the framework guarantees tamper-proof, verifiable, and patient-controlled identity management, which is crucial for all subsequent consent and data-sharing processes.

----

2) Step 2: Healthcare Provider Adds Records

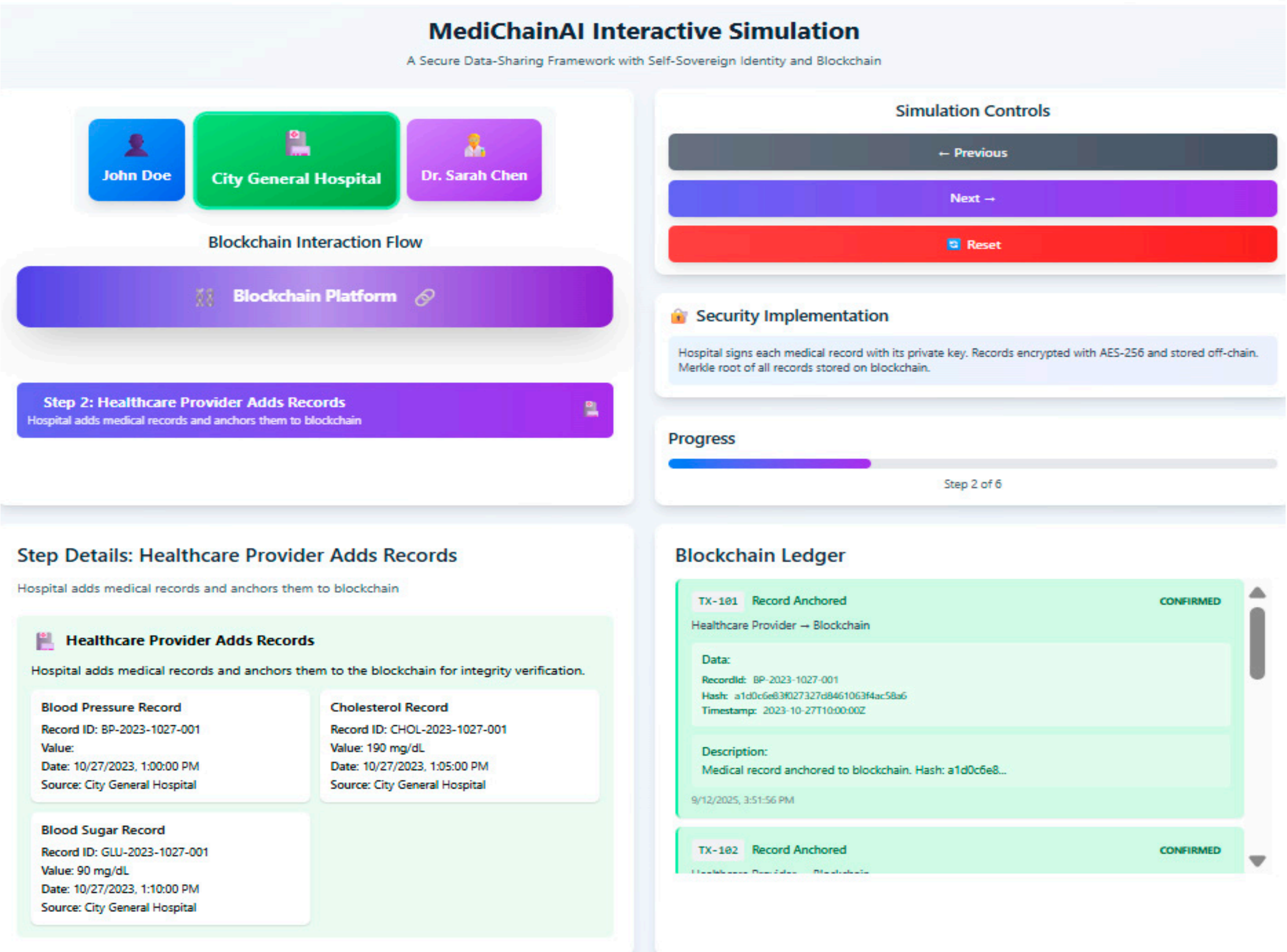

Figure S2: Healthcare Provider Adds Records

This screen shows the hospital's role in contributing verified medical data to the Blockchain through MediChainAI.

- **Process flow:**
  - The healthcare provider generates and signs medical records using its private key.
  - Each record is encrypted with AES-256 and stored off-chain for scalability and privacy.
  - The Merkle root of all records is anchored to the Blockchain, ensuring integrity and tamper-evidence.
- **Interface features:**
  - Security Implementation panel describes how AES-256 encryption and signing safeguard the records.
  - Blockchain Ledger confirms anchoring transactions with unique transaction IDs (e.g., TX-121, TX-122), hashes, and timestamps.
- **Record examples:**
  - *Blood Pressure Record* (120/80, timestamped and hospital-signed)
  - *Cholesterol Record* (190 mg/dL)
  - *Blood Sugar Record* (90 mg/dL)
- **Significance:**

  This step demonstrates how healthcare providers ensure the authenticity, provenance, and immutability of medical records. By anchoring hashed data and storing detailed metadata, the framework guarantees that AI/ML systems later consume only verified and trustworthy health data.

----

3) Step 3: Researcher Requests Access

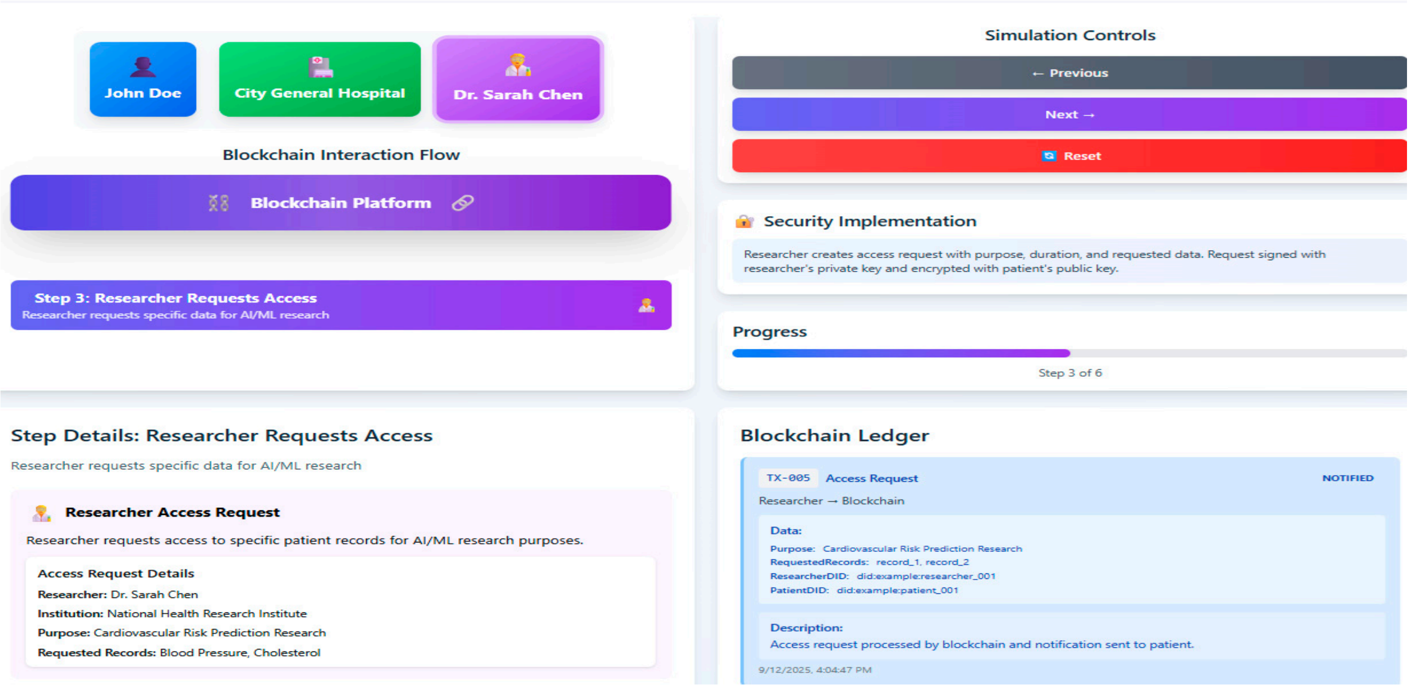

Figure S3: Researcher Requests Access

This screen demonstrates how researchers formally request permission to use specific patient data for AI/ML-driven research within MediChainAI.

- **Process flow:**
  - The researcher (Dr. Sarah Chen) submits a request to access specific medical records.

- The request includes purpose, institution, requested data, and duration of use.
- The request is signed with the researcher's private key and encrypted with the patient's public key, ensuring authenticity and confidentiality.
- The Blockchain processes the request and generates a transaction record (TX-085), with status NOTIFIED, meaning the patient has been alerted for consent.

- **Interface features:**
  - Security Implementation panel explains cryptographic protections.
  - Blockchain Ledger displays detailed request metadata, including:
    - Purpose: Cardiovascular Risk Prediction Research
    - Requested Records: Blood Pressure, Cholesterol
    - ResearcherDID and PatientDID identifiers
  - Notification mechanism ensures the patient is informed before data access proceeds.
- **Significance:**

  This step highlights how MediChainAI enforces fine-grained, consent-driven data access. Researchers cannot bypass patient authorization; instead, every access request is transparent, auditable, and cryptographically secured. This ensures that AI/ML models are trained only on ethically obtained and patient-approved data.

----

4) Step 4: Patient Grants Consent via Smart Contract

This screen shows how the patient exercises direct control over their health data by granting consent through a blockchain-based smart contract.

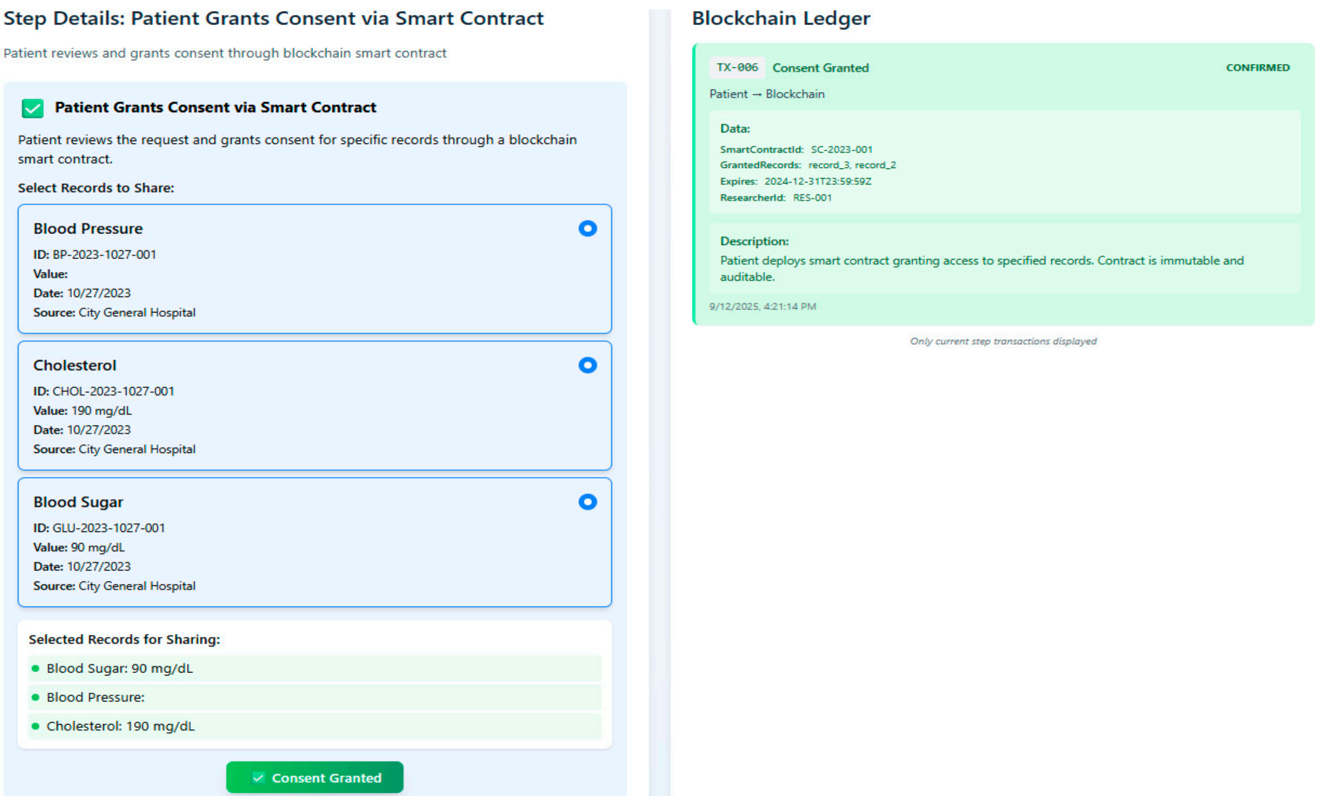

Figure S4: Patient Grants Consent via Smart Contract

- **Process flow:**

- The patient reviews the researcher's request and selects which medical records to share.
- In this screen, the patient authorizes access to all three options:
- Blood Pressure
- Cholesterol
- Blood Sugar
- Once confirmed, a smart contract is deployed on the Blockchain to encode the patient's consent.
- The Blockchain ledger (TX-086) records the granted consent, including:
- *Smart Contract ID*
- *Granted Records* (record_1, record_2, record_3)
- *Expiration date/time* of consent
- *Researcher ID*
- **Interface features:**
- The consent interface provides a clear view of records available, selected records, and their details (ID, value, date, source).
- The Blockchain Ledger confirms the consent transaction with status *CONFIRMED*, ensuring immutability and auditability.
- The "Consent Granted" button finalizes the process, anchoring the authorization on-chain.
- **Significance:**

  This step illustrates fine-grained, auditable consent management. By using smart contracts, MediChainAI ensures that:
- Patients remain the ultimate decision-makers.
- Consent is transparent, revocable, and time-bound.
- Access is enforceable by Blockchain logic, eliminating unauthorized use.

This mechanism aligns with GDPR/HIPAA compliance by embedding privacy, accountability, and patient autonomy directly into the data-sharing infrastructure.

----

5) Researcher Access Data with Verification

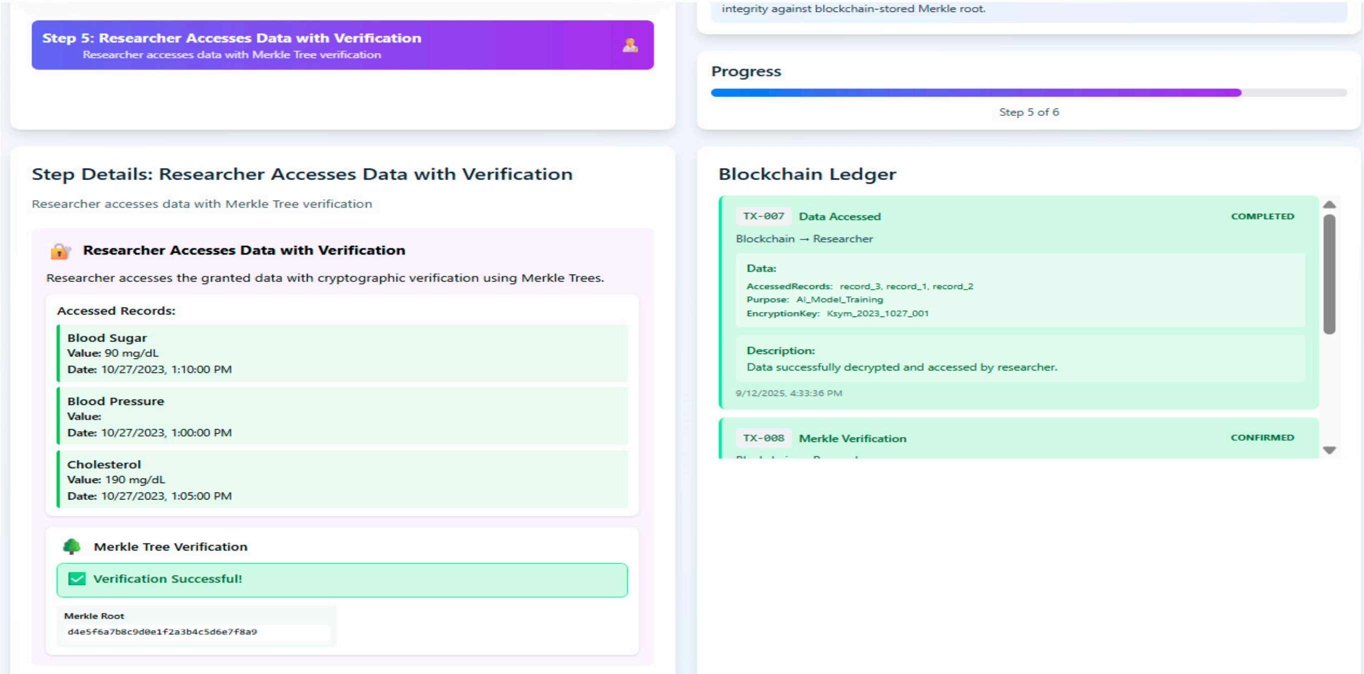

Figure S5: Researcher Access Data with Verification

This screen shows how a researcher securely accesses patient-approved data and verifies its authenticity using **Merkle Trees**.

- **Process flow:**
  - The researcher retrieves the authorized records from the Blockchain-enabled system.
  - Access is cryptographically validated using a Merkle proof against the stored Merkle root.
  - In this case, the researcher successfully accessed and verified:
    - Blood Sugar (90 mg/dL)
    - Blood Pressure
    - Cholesterol (190 mg/dL)
  - The Blockchain ledger logs two key transactions:
    - TX-087 Data Accessed (Completed): confirms decryption and retrieval of records for AI/ML training.
    - TX-088 Merkle Verification (Confirmed): ensures the accessed records are authentic, unaltered, and part of the original dataset.
- **Interface features:**
  - Security Implementation panel notes the Merkle verification process.
  - Merkle Tree Verification box displays a successful validation with the Merkle Root value.
  - Blockchain Ledger provides auditability with transaction IDs, encryption key, and purpose (AI Model Training).
- **Significance:**

  This step illustrates how MediChainAI guarantees data integrity and trust. Using Merkle Trees, researchers can verify subsets of patient data without requiring full dataset disclosure. This ensures that AI/ML models are trained only on authentic, tamper-proof medical data, while preserving patient privacy.

----

6) AI/ML Model Training with Privacy

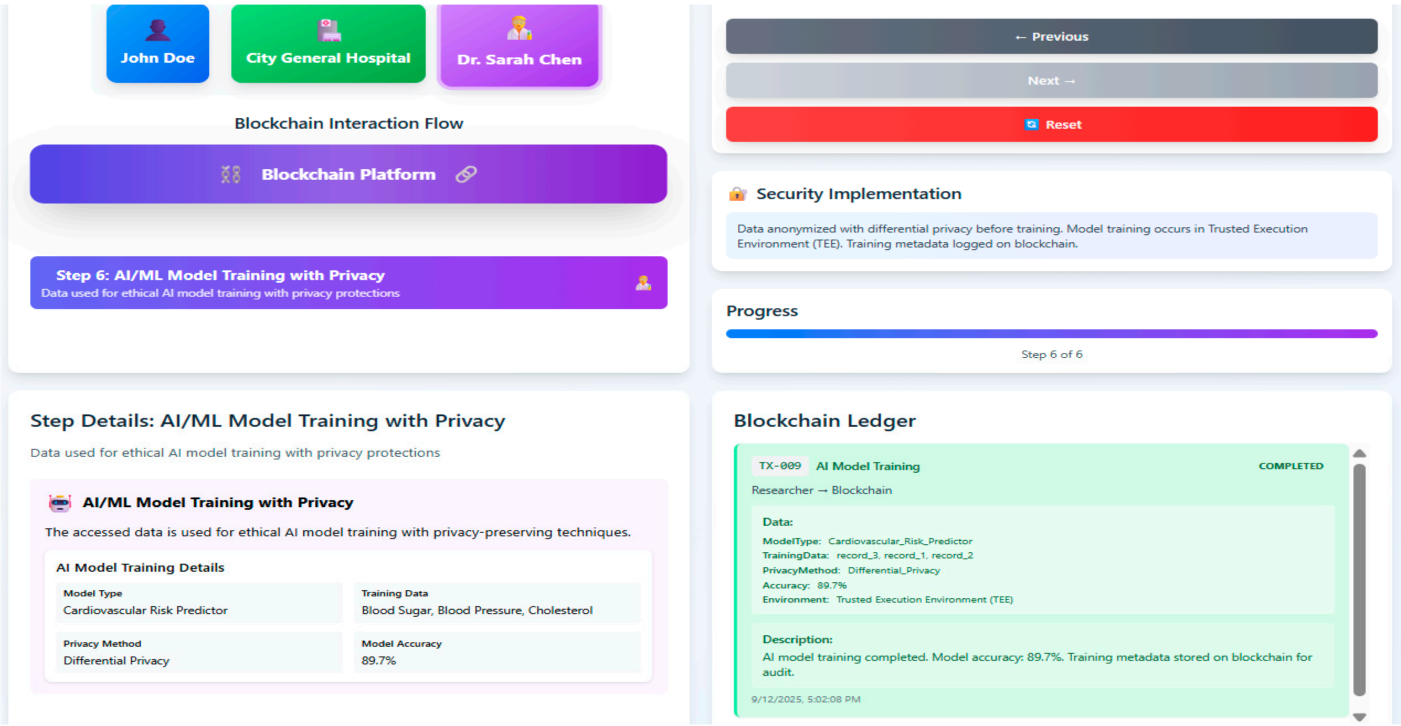

Figure S6: AI/ML Model Training with Privacy

This screen illustrates the final stage of the MediChainAI workflow, where patient-approved and verified data is used for privacy-preserving AI/ML model training.

- **Process flow:**
  - The accessed data (Blood Sugar, Blood Pressure, Cholesterol) is anonymized using Differential Privacy before training.
  - Training is executed inside a Trusted Execution Environment (TEE) to ensure isolation from external access.
  - The Blockchain records metadata about the training process, including model type, privacy method, and accuracy.
- **Interface features:**
  - Security Implementation panel highlights differential privacy and TEE usage.
  - AI Model Training Details include:
    - *Model Type:* Cardiovascular Risk Predictor
    - *Training Data:* Blood Sugar, Blood Pressure, Cholesterol
    - *Privacy Method:* Differential Privacy
    - *Model Accuracy:* 89.7%
  - Blockchain Ledger (TX-009) logs model-training completion, accuracy results, and environment used (*TEE*).

- **Significance:**
  This step demonstrates how MediChainAI enforces ethical AI integration. By embedding privacy-preserving methods and recording training metadata on Blockchain, the

framework ensures that AI/ML outcomes are trustworthy, auditable, and compliant with data protection regulations. Patients can be confident that their data not only remains secure but also contributes responsibly to medical innovation.
